# Molecular cytogenetic analysis of 11 new breast cancer cell lines

**DOI:** 10.1038/sj.bjc.6695007

**Published:** 1999-12

**Authors:** F Forozan, R Veldman, C A Ammerman, N Z Parsa, A Kallioniemi, O-P Kallioniemi, S P Ethier

**Affiliations:** Cancer Genetics Branch, National Human Genome Research Institute, National Institutes of Health, Bethesda, MD 20892-4470, USA; Department of Radiation Oncology, Division of Radiation and Cancer Biology, University of Michigan Medical School, 7312 Cancer Center, 1500 E. Medical Center Dr, Ann Arbor, MI 48109-0948, USA

**Keywords:** molecular cytogenetics, FISH, CGH, chromosomal aberrations, oncogene amplification

## Abstract

We describe a survey of genetic changes by comparative genomic hybridization (CGH) in 11 human breast cancer cell lines recently established in our laboratory. The most common gains took place at 8q (73%), 1q (64%), 7q (64%), 3q (45%) and 7p (45%), whereas losses were most frequent at Xp (54%), 8p (45%), 18q (45%) and Xq (45%). Many of the cell lines displayed prominent, localized DNA amplifications by CGH. One-third of these loci affected breast cancer oncogenes, whose amplifications were validated with specific probes: 17q12 (two cell lines with ERBB2 amplifications), 11q13 (two with cyclin-D1), 8p11–p12 (two with FGFR1) and 10q25 (one with FGFR2). Gains and amplifications affecting 8q were the most common genetic alterations in these cell lines with the minimal, common region of involvement at 8q22–q23. No high-level MYC (at 8q24) amplifications were found in any of the cell lines. Two-thirds of the amplification sites took place at loci not associated with established oncogenes, such as 1q41–q43, 7q21–q22, 7q31, 8q23, 9p21–p23, 11p12–p14, 15q12–q14, 16q13–q21, 17q23, 20p11–p12 and 20q13. Several of these locations have not been previously reported and may harbour important genes whose amplification is selected for during cancer development. In summary, this set of breast cancer cell lines displaying prominent DNA amplifications should facilitate discovery and functional analysis of genes and signal transduction pathways contributing to breast cancer development. © 1999 Cancer Research Campaign


*British Journal of Cancer* (1999) **81**(8), 1328–1334
© 1999 Cancer Research Campaign
Article no. bjoc.1999.0848

### Molecular cytogenetic analysis of 11 new breast cancer cell lines

**F Forozan[1], R Veldman[1], CA Ammerman[2], NZ Parsa[1], A Kallioniemi[1], O-P Kallioniemi[1] and SP Ethier[2]**

[1]Cancer Genetics Branch, National Human Genome Research Institute, National Institutes of Health, Bethesda, MD 20892-4470, USA; [2]Department of Radiation Oncology, Division of Radiation and Cancer Biology, University of Michigan Medical School, 7312 Cancer Center, 1500 E. Medical Center Dr, Ann Arbor, MI 48109-0948, USA

**Summary** We describe a survey of genetic changes by comparative genomic hybridization (CGH) in 11 human breast cancer cell lines recently established in our laboratory. The most common gains took place at 8q (73%), 1q (64%), 7q (64%), 3q (45%) and 7p (45%), whereas losses were most frequent at Xp (54%), 8p (45%), 18q (45%) and Xq (45%). Many of the cell lines displayed prominent, localized DNA amplifications by CGH. One-third of these loci affected breast cancer oncogenes, whose amplifications were validated with specific probes: 17q12 (two cell lines with ERBB2 amplifications), 11q13 (two with cyclin-D1), 8p11–p12 (two with FGFR1) and 10q25 (one with FGFR2). Gains and amplifications affecting 8q were the most common genetic alterations in these cell lines with the minimal, common region of involvement at 8q22–q23. No high-level MYC (at 8q24) amplifications were found in any of the cell lines. Two-thirds of the amplification sites took place at loci not associated with established oncogenes, such as 1q41–q43, 7q21–q22, 7q31, 8q23, 9p21–p23, 11p12–p14, 15q12–q14, 16q13–q21, 17q23, 20p11–p12 and 20q13. Several of these locations have not been previously reported and may harbour important genes whose amplification is selected for during cancer development. In summary, this set of breast cancer cell lines displaying prominent DNA amplifications should facilitate discovery and functional analysis of genes and signal transduction pathways contributing to breast cancer development. © 1999 Cancer Research Campaign

**Keywords:** molecular cytogenetics; FISH; CGH; chromosomal aberrations; oncogene amplification

Development and progression of breast cancer is associated with the accumulation of genetic changes involving oncogenes, tumour suppressor genes and several other genes. In breast cancer, gene amplifications often involve *ERBB2* (at 17q12), *cyclin-D1* (11q13) and *MYC* (8q24). In addition, other genes, such as *FGFR1* (8p11), (10q25) and *IGFR1* (15q25) may also undergo amplification (Devilee et al, 1994). Recently, numerous additional chromosomal regions of increased copy number, such as 17q23 and 20q13, have been reported by comparative genomic hybridization (CGH) and microdissection (Guan et al, 1994; Kallioniemi et al, 1994; Tanner et al, 1994). These loci may also harbour genes with an important role in breast cancer progression. In a similar fashion, mutations or inactivations of several tumour suppressor genes, such as *p53, p16* and *RB1*, have been reported (Devilee et al, 1994; Geradts et al, 1996; Li et al, 1997), but loss of heterozygosity and CGH analyses suggest several other chromosomal regions, such as 1p, 3p, 6q, 8p and 16q (Devilee et al, 1994), where additional putative tumour suppressor genes may reside. Overall, many chromosomal regions appear to be involved in breast cancer development and progression, but in most cases, the genes implicated in these rearrangements remain unknown.

Cancer cell lines provide an important resource for cancer gene discovery as well as for functional studies. Several breast cancer cell lines exist, but information on their origin, as well as their genetic and molecular characteristics are very fragmentary. Over the past several years, our laboratory has developed and optimized methods for the culture of normal human mammary epithelial cells of the luminal lineage (Ethier et al, 1990, 1993). We have also established a panel of 11 SUM-human breast cancer cell lines, which are maintained continuously in the laboratory. The establishment of some of these cell lines, including SUM-44, SUM-52, and SUM-102 has been described previously (Ethier et al, 1993, 1996; Sartor et al, 1997). In addition, studies on Stat3 activation and focal adhesion kinase activation in human breast cancer cells that made use of the above lines, as well as the SUM-149, SUM-1315, SUM-159, SUM-185 and SUM-190 cell lines, have been presented elsewhere (Garcia et al, 1997; Flanagan et al, 1999; Ignatoski et al, 1999).

Here, we performed a molecular cytogenetic survey of genetic changes by CGH to study to what extent these 11 cell lines resemble primary human breast carcinomas, as well as to evaluate whether these cell lines contain characteristic genetic changes that could facilitate cancer gene discovery.

*Received 1 March 1999*
*Revised 26 May 1999*
*Accepted 28 May 1999*

*Correspondence to:* SP Ethier

### MATERIALS AND METHODS

### Isolation and culture of human breast cancer cell lines

The isolation and culture of cell lines designated SUM-44, SUM-52 and SUM-102 have been described in detail (Ethier et al, 1993, 1996; Sartor et al, 1997). More recently, our laboratory has developed seven other human breast cancer cell lines from primary tumours (PT), chest wall recurrences (CWN) and pleural effusion (PE) metastases (Table 1). In addition, one cell line was developed from a highly invasive breast cancer specimen that had been

**Table 1** Preparation of the SUM breast cancer cell lines: origin of the tissue, prior chemotherapy given to the patients, and media requirements for sustained in vitro growth

| Cell line | Breast cancer specimen | Prior chemotherapy | Media supp.[a] |
|---|---|---|---|
| SUM-44PE | Pleural effusion | + | SFIH |
| SUM-52PE | Pleural effusion | + | 5%IH |
| SUM-102PT | Intraductal carcinoma/micro-invasion | + | SFIHE |
| SUM-1315M02 | Skin metastasis of inflitrating ductal carcinoma | + | SFIE |
| SUM-149PT | Invasive ductal carcinoma (inflammatory) | + | 5%IH |
| SUM-159PT | Anaplastic carcinoma | – | 5%IH |
| SUM-185PE | Pleural effusion | + | 5%IH |
| SUM-190PT | Invasive ductal breast carcinoma (Inflammatory) | + | SFIH |
| SUM-206CWN | Chest wall recurrence of invasive ductal carcinoma | + | 5%IHP |
| SUM-225CWN | Chest wall recurrence of ductal carcinoma in situ | – | 5%IH |
| SUM-229PE | Pleural effusion | + | 5%IH |

[a]5%, 5% fetal bovine serum; SF, serum-free; I, insulin; H, hydrocortisone; E, epidermal growth factor, P, progestrone.

grown for two transplant generations in immunodeficient mice (MO2) before being explanted into culture. The cells derived from these xenografts were kindly provided by Dr D Schwartzentruber of the Surgery Branch, NCI. The newly developed cell lines have been designated: SUM-149, SUM-1315, SUM-159, SUM-185, SUM-190, SUM-206, SUM-225 and SUM-229. SUM-149 and SUM-190 were derived from patients with inflammatory breast cancer.

Nine of the 11 patients had received chemotherapy prior to sampling. All cell lines are immortal, and express luminal cytokeratins consistent with their origin from luminal breast epithelial cells. The cell lines were isolated and grown in a variety of media that were previously optimized for the culture of normal human breast epithelial cells (Mahacek et al, 1993; Ethier et al, 1996). All culture media were prepared from a base medium of Ham's F-12 (Table 1). Some media were supplemented with 5% fetal bovine serum (FBS). Serum-free media were supplemented with 0.1% bovine serum albumin (BSA), sodium selenite, 3,3′,5-triiodo-L-thyronine, transferrin and ethanolamine as previously described (Ethier et al, 1993, 1996; Sartor et al, 1997). Several of the cell lines (SUM-52, 149, 159, 185, 225 and 229) were routinely cultured in Ham's F-12 supplemented with 5% FBS, insulin (5 μg $ml^{-1}$) and hydrocortisone (1 μg $ml^{-1}$, 5% IH medium). SUM-1315 cells were cultured in serum-free medium with insulin and EGF (10 ng $ml^{-1}$). SUM-190 cells were routinely cultured in serum-free medium with insulin and hydrocortisone, but were originally isolated in a more complex medium supplemented with epidermal growth factor (EGF) and lysophosphatidic acid (10 mM), the latter of which was required for initial growth of the cells. SUM-206 cells were cultured in 5% IH medium supplemented with progesterone (100 ng $ml^{-1}$). These cells, like SUM-102 cells described previously (Sartor et al, 1997), were responsive to exogenous progesterone for in vitro growth. Thus, this panel of human breast cancer cell lines showed different requirements for exogenous hormones and growth factors (Table 1).

All 11 breast cancer cell lines isolated were immortal in culture, and were karyotypically abnormal. Information on the in vitro morphology and karyotype of the cell lines can be found at http://p53.cancer.med.umich.edu/clines/clines.html. Detailed methods for the growth of each of these cell lines can be found at http://p53.cancer.med.umich.edu/clines/elab/ethier.html. In addition, these cells uniformly expressed the luminal cytokeratins 8 and 18, and all cell lines except the SUM-102 line expressed keratin 19 as determined by immunocytochemistry.

### Comparative genomic hybridization

CGH was carried out on the 11 breast carcinoma cell lines essentially as described previously (Karhu et al, 1997; Tirkkonen et al, 1998) with some modifications. Briefly, genomic cell line (test) and normal female (reference) DNAs were labelled by nick translation incorporating either SpectrumGreen or SpectrumRed dUTPs (Vysis Inc., Downers Grove, IL, USA). Two hundred nanograms of green-labelled cell line DNA, 100 ng of red-labelled normal reference DNA, and 10 μg of unlabelled Cot-1 DNA were hybridized to denatured normal peripheral blood metaphase slides. After a 2-day hybridization at 37°C, the slides were washed and counterstained with DAPI in an antifade medium. Image acquisition was performed with a digital image analysis system using a Zeiss Axiophot microscope and a Photometrics CCD camera. The system was controlled by the IPLab Spectrum software for PowerMacintosh. After acquisition of digital images on wavelengths matching the DAPI, SpectrumGreen and SpectrumRed emissions, green to red ratio profiles were quantitated with Quips XL program (Vysis Inc.). Green and red intensities were normalized so that the average green to red ratio in each metaphase was set to 1.0. Normal male versus female hybridizations were used as negative controls, and for ensuring the linearity of the hybridization (Karhu et al, 1997). MPE-600 breast cancer cell line with known aberrations was used as a positive control. Chromosomal regions where CGH ratios exceeded 1.2 were considered as gained, and those regions where the ratio was less than 0.8 as lost. High-level amplifications were defined as small regions (1–3 chromosomal bands wide) with highly elevated ratio (ratio > 1.4). In the case of very small regions of amplification, the average ratio profile from multiple metaphases may not reach this cut-off value, since the peak ratio may take place at different locations in the different metaphases as a result of the non-linear stretching of metaphase chromosomes. Therefore, ratio cut-offs were evaluated from individual profiles (low-level amplification).

### Fluorescent in situ hybridization

Dual colour fluorescent in situ hybridization (FISH) analysis was done on interphase and metaphase slides prepared from the cell lines as described before (Tanner et al, 1994). To facilitate FISH analysis of interphase nuclei, multi-spot slides were made, each containing nuclei from all the 11 cell lines. The slides were

© 1999 Cancer Research Campaign

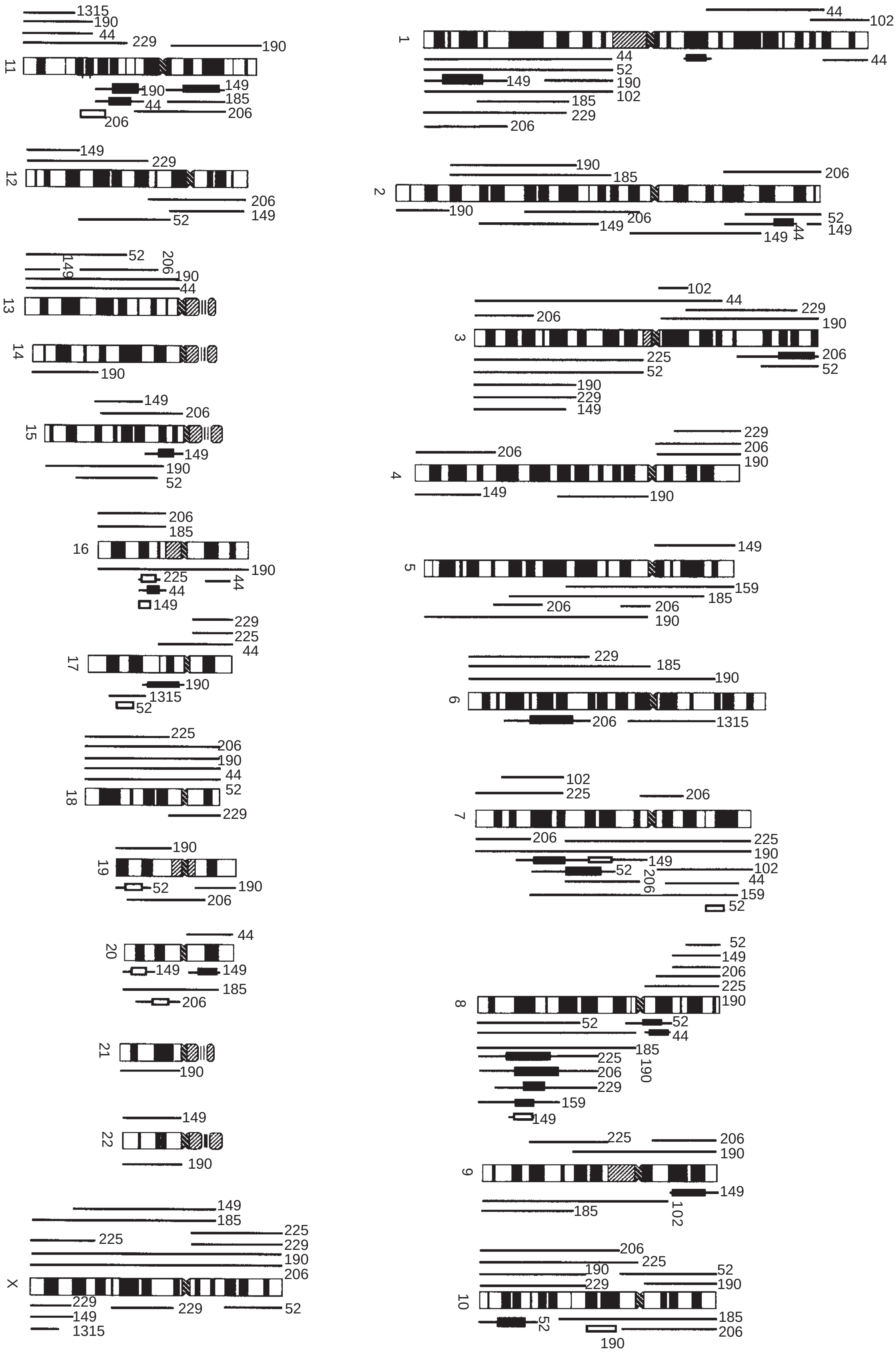

**Figure 1** Summary ideogram of gains (right) and losses (left) of chromosomal regions seen by CGH in 11 SUM breast cancer cell lines. Black boxes represent regions of high level amplification by CGH, and open boxes other small regions of low level amplification

 *© 1999 Cancer Research Campaign*

**Table 2** Most common gains and losses in the SUM panel of breast cancer cell lines by CGH

| Gains | | Losses | |
|---|---|---|---|
| Chromosome region | (%) | Chromosome region | (%) |
| 8q (q22–q24.1)[a] | 73 | Xp (cen-p11.3) | 54 |
| 1q (q32) | 64 | 8p (p22–p23) | 45 |
| 7q (q21–q22) | 64 | 18q (q12–q22) | 45 |
| 3q (q24–q29) | 45 | Xq (p24–p25) | 45 |
| 7p (p12–p21) | 45 | 10q (q22–q26) | 36 |
| 5q (q11.2–q12) | 36 | 11q (q23–q25) | 36 |
| 2p (p21) | 27 | 13q (q21–q22) | 36 |
| 11p (cen-p14) | 27 | 13q (q32–q34) | 36 |
| 11q (q13) | 27 | 18p | 36 |
| 15q (q15) | 27 | 4p (p14–p16) | 27 |
| 20q (q13) | 27 | 3p (p12) | 27 |
| Xq (q27–q28) | 27 | 3p (p13–p14) | 27 |
| | | 6q (q21–q27) | 27 |
| | | 17p | 27 |

[a]The minimal common chromosomal region of involvement is indicated in parenthesis

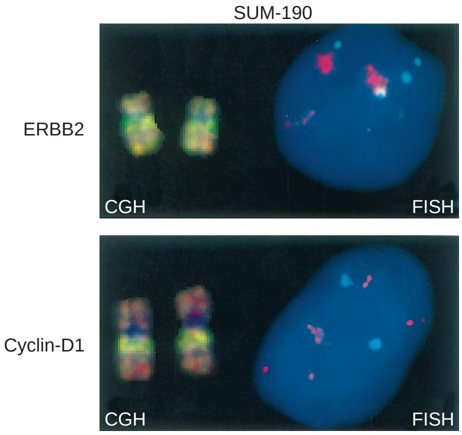

**Figure 2** Detection of amplifications of the *ERBB2* and *cyclin-D1* genes in the SUM panel of breast cancer cell lines by CGH and FISH. SUM-190 had both *ERBB2* and *cyclin-D1* gene amplification. The same amplifications were visible as increased green fluorescent intensity at 17q12 and 11q13 by CGH and as increased number of signals in interphase FISH. The *ERBB2* and *cyclin-D1* probes were visualized in the cell line nuclei in red colour, whereas the corresponding chromosome centromere probes (for chromosomes 17 and 11 respectively) were visualized in green colour hybridized with SpectrumOrange labelled probes for *cyclin-D1*, *ERBB2*, and *MYC* with the corresponding SpectrumGreen-labelled centromeric probe for chromosomes 11, 17 and 8 as reference probes (Vysis Inc.). Since the cell lines were genetically rather homogeneous, we scored 20 non-overlapping nuclei with intact morphology based on DAPI counterstaining to determine the mean copy number of the gene probes per cell as well as the copy number relative to the chromosome specific reference probe. Greater than three-fold increase in the copy number of the test probe over that of the reference probes was considered to represent significant gene amplification.

### Southern blot

For Southern blot analysis, genomic DNA was isolated from confluent monolayers of cells. Fifteen micrograms of *Eco*RI- or *Hin*dIII-digested DNA was loaded onto 0.8% agarose gels, separated by electrophoresis and transferred onto Immobilon-N membrane (Millipore Corp., Bedford, MA, USA). Ethidium bromide-stained agarose gels were photographed prior to transfer to ensure equal loading of the gel. The transfer membrane was UV-cross-linked, prehybridized overnight and then hybridized with $^{32}$P-labelled probes specific for FGFR1 and FGFR2. The specific activity of the probes was always greater than $1 \times 10^8$ cpm $ml^{-1}$. Hybridization proceeded at 37°C for 24 h. Following hybridization, the membrane was washed twice in 2 × standard saline citrate (SSC), 0.2% sodium dodecyl sulphate (SDS) for 10 min at room temperature, once in 1 × SSC, 0.2% SDS for 15 min at room temperature, and once in 0.5 × SSC, 0.2% SDS for 20 min at 50°C. The membrane was then exposed to X-ray film for autoradiography.

### RESULTS

### Gains and losses of DNA sequences by CGH

All 11 breast cancer cell lines displayed copy number alterations by CGH as expected based on the presence of abnormal karyotypes in G-banding analysis. There were, on average, 14 genetic changes in each cell line (range from 4 to 29) with 6 losses (range from 0 to 14) and 8 gains (range 3 to 15) per cell line. A summary of the different regions of gains and losses is shown in an ideogram format in Figure 1. The minimal common regions for the most frequent copy number gains were: 8q22–q24.1 (73% of the cases), 1q32 (64%), 7q21–q22 (64%), 3q24–q29 (45%) and 7p12–p21 (45%). The most common losses were: Xcen-p11.3 (54%), 8p22–p23 (45%), 18q12–q22 (45%), and Xq24–q25 (45%) (Table 2).

Most of the gains of DNA sequence copy number were of low magnitude and affected large regions. However, many cell lines also displayed informative small, localized regions of increased DNA sequence copy numbers, often of considerable magnitude (ratios from 1.4 to 2.1). Since these may pinpoint locations of important genes, we performed a separate analysis of such localized copy number increases.

### DNA amplifications of known oncogene loci

Many of the amplifications seen by CGH-affected chromosomal regions where genes, previously shown to be amplified in breast cancer, reside. These regions included the *ERBB2* locus at 17q12, *MYC* at 8q24.1, *cyclin-D1* at 11q13, *FGFR1* at 8p12, and *FGFR2* at 10q25. Amplifications of these five genes were tested using specific probes by FISH (ERBB2, MYC, Cyclin-D1) (Figure 2) or Southern blot (for FGFR1 and FGFR2) as summarized in Table 3. With the exception of the MYC locus (see below), a peak in the CGH profile at these chromosomal sites reflected a high-level amplification of the known target genes at these chromosomal regions. For example, SUM-52 cells had very prominent peaks by CGH at 8p11–p12 and at 10q24–q25, and Southern analysis confirmed high-level amplifications of both FGFR1 and FGFR2. As expected, analyses with specific probes also revealed amplifications that did not result in a peak of ratio profiles in CGH. A high level amplification of ERBB-2 in the SUM-225 cell line did not lead to a significant increase of the copy number ratio at 17q12.

© 1999 Cancer Research Campaign

**Table 3** Amplification of known oncogenes in the SUM panel of breast cancer cell lines

| Cell line | Cyclin-D1 | c-MYC | ERBB2 | FGFR1 | FGFR2 |
|---|---|---|---|---|---|
| SUM-44 | + | – | – | + | – |
| SUM-52 | + | – | – | + | + |
| SUM-102 | – | – | – | – | – |
| SUM-1315 | – | – | – | – | – |
| SUM-149 | – | – | – | – | – |
| SUM-159 | – | – | – | – | – |
| SUM-185 | – | – | – | – | – |
| SUM-190 | + | – | + | – | – |
| SUM-206 | – | – | – | – | – |
| SUM-225 | – | – | + | – | – |
| SUM-229 | – | – | – | – | – |

The + and – signs represent the presence or absence of amplification, respectively, detected by FISH (for cyclin-D1, c-MYC, and ERBB2) or Southern (for FGFR1 and FGFR2).

### DNA amplifications at other chromosomal loci

CGH analysis of the 11 breast cancer cell lines also resulted in the detection of several sites of highly increased DNA sequence copy number in regions not known to harbour breast cancer oncogenes (Figure 3). Up to 69% of all DNA amplifications were localized at such 'new' regions of amplification. These regions included 1q41–q43, 7q21–q22, 7q31, 8q23, 9p21–p23, 11p12–p14, 15q12–q14, 16q13–q21, 17q23, 20p11–12, and 20q13. Gain of 8q was the most common abnormality seen by CGH, and in four cases high-level amplifications were seen (SUM-225, 229, 159, 206). The minimal region of amplification was at 8q22–q23, slightly proximal to the MYC locus at 8q24.1. In agreement with this observation, none of the cell lines displayed high-level amplifications of MYC by FISH. However, SUM-159 and SUM-225 did have approximately twofold increases of MYC copy number as determined by FISH.

### DISCUSSION

The data presented here on the genetic changes of the 11 newly established breast cancer cell lines provide a useful resource and starting point for discovery of novel cancer genes and signalling pathways. Several observations indicate that these cell lines resemble primary human breast cancers. First, CGH analyses revealed several recurrent chromosomal gains, such as those at 1q, 3q and 8q, that are also very common in uncultured breast cancers (Ried et al, 1995; Kuukasjarvi et al, 1997; Tirkkonen et al, 1998). Second, many of the losses of chromosomal regions in these cell lines, such as those at 3p, 6q, 8p, 11q, 13q, 17p, 18q and X, have also been previously implicated in both LOH (Devilee et al, 1994) and CGH studies (Ried et al, 1995; Kuukasjarvi et al, 1997; Tirkkonen et al, 1998) of primary breast cancer specimens. Third, the spectrum and frequency of amplifications involving the known oncogene loci (ERBB2, cyclin-D1, FGFR1 and FGFR2) in these cell lines were similar to those previously reported for uncultured human breast cancers (Adnane et al, 1991; Devilee et al, 1994).

The CGH data indicated the presence of several characteristic high level DNA amplifications in these cell lines. The majority of these involved chromosomal sites distinct from those of known oncogenes. DNA amplification is known to be a predominant mechanism for oncogene activation in many solid tumours. It can be suspected that the other regions found here also contain genes that are important in the development and progression of breast cancer. This issue is supported by recent molecular studies of the 20q region, the only amplification site discovered by CGH that has so far been extensively studied. Many potential target genes, including serine-threonine kinase *BTAK*/*Aurora II* (Sen et al, 1997; Bischoff et al, 1998), steroid receptor co-activator *AIB1* (Anzick et al, 1997), MYBL2 oncogene (Kononen et al, 1998) *PTPN1* phosphotyrosine phosphorylase (Tanner et al, 1996), as well as ZNF217 and NABC1 (Collins et al, 1998) genes have been found to be involved in DNA amplifications at 20q.

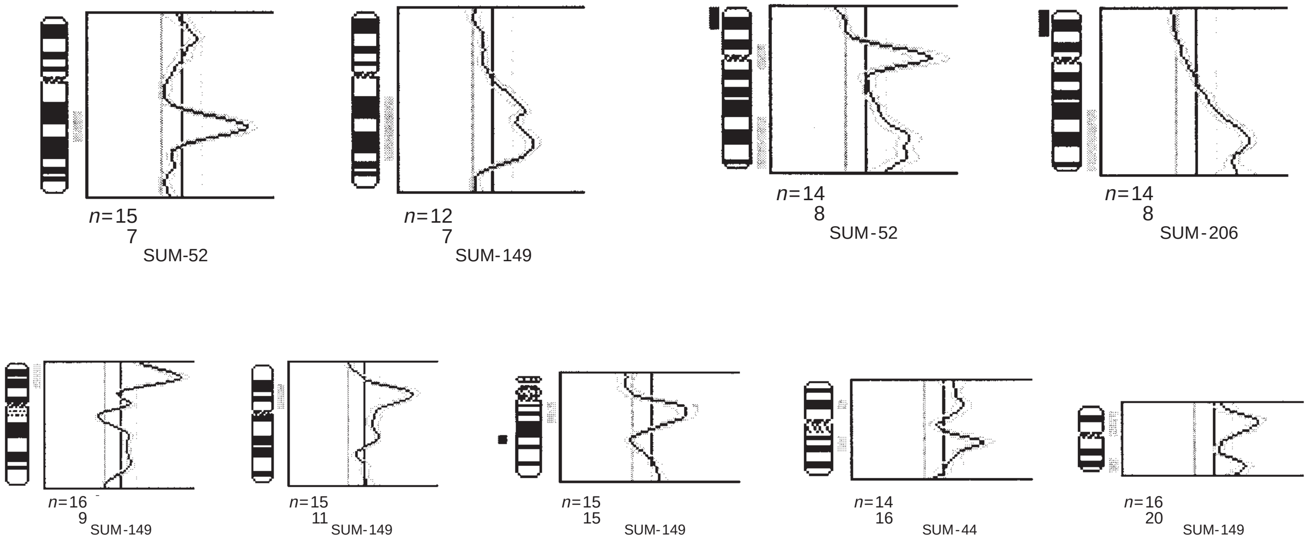

**Figure 3** Examples of CGH profiles illustrating prominent gains and amplifications involving chromosomal sites such as 7q21–q22, 7q31, 8q23, 9p21–p23, 11p12–p14, 15q12–q14, 16q13–q21 and 20p11–p12. The green to red fluorescent intensity profiles are shown along with ± 1 s.d. For each profile, the vertical line in the middle is the ratio value of 1.0, the line to the left represents a ratio cut-off value of 0.8, and the line to the right 1.2. Chromosome ideograms displayed next to the profile have regions of gain (right) and loss (left) marked as bars

 *© 1999 Cancer Research Campaign*

Amplification sites that were seen in this panel of cell lines, and where candidate genes have not been defined include 7q21–q22, 7q31, 8q23, 15q12–q14, 16q13–q21 and 17q23 (Figure 3). It is likely that these sites are also of importance to breast cancer progression and harbour new candidate genes. Many of these amplifications, such as 7q31, often also appear in vivo in uncultured cancers affecting organs other than breast, such as brain (Wullich et al, 1993; Fischer et al, 1995) and gastric cancer (Houldsworth et al, 1990). The 8q23–q24 chromosomal region was the most common site of increased copy number in this panel of cancer cell lines and was present in eight of the 11 cell lines. Many gains involved the whole chromosome arm, but there were also several localized increases of copy number at 8q23, a site slightly proximal to the *MYC* oncogene locus at 8q24.1. Since FISH analyses indicated only a twofold increase of the copy number of *MYC* in two cell lines, and no increase in any of the other ones, it is likely that genes other than *MYC* are the primary targets for the selection of 8q gains and 8q23–q24 amplifications in these cells. Further studies of this chromosomal site are important, as gain of the 8q arm is one of the most common genetic aberrations in breast cancer and several other tumour types (Forozan et al, 1997).

These results show that this panel of breast cancer cell lines could be useful for identification of cancer genes from the recurrent sites of chromosomal aberrations mapped in this study. In addition, they may facilitate studies of growth regulatory mechanisms that distinguish breast cancer cells from normal human breast epithelial cells.

Overall, these phenotypically and genetically characterized cell lines may serve as a starting point for the discovery of previously unknown signalling pathways, as well as help in the functional analysis of known signal transduction pathways operative in human breast cancer cells. In addition, several high-level amplification sites in these cell lines are likely to be useful as a starting point for cancer gene discovery.

### ACKNOWLEDGEMENTS

Part of the work was supported by grant number DAMD17-94-J4382, Department of Defense, breast cancer programme. The authors wish to thank Darryl Leja for help with the technical illustrations.

### REFERENCES

Adnane J, Gaudray P, Dionne CA, Crumley G, Jaye M, Schlessinger J, Jeanteur P, Bimbaum D and Theillet C (1991) BEK and FLG, two receptors to members of the FGF family, are amplified in subsets of human breast cancers. *Oncogene* **6**: 659–663

Anzick SL, Kononen J, Walker RL, Azorsa DO, Tanner MM, Guan XY, Sauter G, Kallioniemi OP, Trent JM and Meltzer PS (1997) AIB1, a steroid receptor coactivator amplified in breast and ovarian cancer. *Science* **277**: 965–968

Bischoff JR, Anderson L, Zhu Y, Mossie K, Ng L, Souza B, Schryver B, Flanagan P, Clairvoyant F, Ginther C, Chan CS, Novotny M, Slamon DJ and Plowman GD (1998) A homologue of *Drosophila aurora* kinase is oncogenic and amplified in human colorectal cancers. *EMBO J* **17**: 3052–3065

Collins C, Rommens JM, Kowbel D, Godfrey T, Tanner M, Hwang SI, Polikoff D, Nonet G, Cochran J, Myambo K, Jay KE, Froula J, Cloutier T, Kuo WL, Yaswen P, Dairkee S, Giovanola J, Hutchinson GB, Isola J, Kallioniemi OP, Palazzolo M, Martin C, Ericsson C, Pinkel D, Albertson D, Li W-B and Gray JW (1998) Positional cloning of ZNF217 and NABC1: genes amplified at 20q13.2 and overexpressed in breast carcinoma. *Proc Natl Acad Sci USA* **95**: 8703–8708

Devilee P and Cornelisse CJ (1994) Somatic genetic changes in human breast cancer. *Biochem et Biophys Acta* **1198**: 113–130

Ethier SP (1996) Human breast cancer cell lines as models of growth regulation and disease progression. *J Mamm Gland Biol Neoplas* **1**: 111–121

Ethier SP, Summerfelt RM, Cundiff KC and Asch BB (1990) The influence of growth factors on the proliferative potential of normal and primary breast cancer-derived human breast epithelial cells. *Breast Cancer Res Treat* **17**: 221–230

Ethier SP, Mahacek ML, Gullick WJ, Frank TJ and Weber BL (1993) Differential isolation of normal luminal mammary epithelial cells and breast cancer cells from primary and metastatic sites using selective media. *Cancer Res* **53**: 627–635

Fischer U, Muller HW, Sattler HP, Feiden K, Zang KD and Meese E (1995) Amplification of MET in gliomas. *Genes Chromosomes Cancer* **12**: 63–65

Flanagan L, VanWeelden K, Ammerman C, Ethier SP and Welsh J (1999) SUM-159PT cells: a novel estrogen independent human breast cancer model system. *Breast Cancer Res Treat* (in press)

Forozan F, Karhu R, Kononen J, Kallioniemi A and Kallioniemi OP (1997) Genome screening by comparative genomic hybridization. *Trends Genet* **13**: 405–409

Garcia R, Yu CL, Hudnall A, Nelson KL, Smithgall T, Fujita DJ, Ethier SP and Jove R (1997) Constitutive activation of STAT3 in fibroblasts transformed by diverse oncoproteins and in breast carcinoma cells. *Cell Growth Diff* **8**: 1267–1276

Geradts J and Wilson PA (1996) High frequency of aberrant p16 (INK4A) expression in human breast cancer. *Am J Pathol* **149**: 15–20

Guan XY, Meltzer PS, Dalton WS and Trent JM (1994) Identification of cryptic sites of DNA sequence amplification in human breast cancer by chromosome microdissection. *Nat Genet* **8**: 155–161

Houldsworth J, Cordon-Cardo C, Ladanyi M, Kelsen DP and Chaganti RS (1990) Gene amplification in gastric and esophageal adenocarcinomas. *Cancer Res* **50**: 6417–6422

Ignatoski KM and Ethier SP (1999) Constitutive activation of pp125fak in newly isolated human breast cancer cell lines. *Breast Cancer Res Treat* (in press)

Kallioniemi A, Kallioniemi OP, Piper J, Tanner M, Stokke T, Chen L, Smith HS, Pinkel D, Gray JW and Waldman FM (1994) Detection and mapping of amplified DNA sequences in breast cancer by comparative genomic hybridization. *Proc Natl Acad Sci USA* **91**: 2156–2160

Karhu R, Kähkönen M, Kuukasjärvi T, Pennanen S, Tirkkonen M and Kallioniemi O (1997) Quality control of CGH: impact of metaphase chromosomes and the dynamic range of hybridization. *Cytometry* **28**: 198–205

Kononen J, Bubendorf L, Kallioniemi A, Barlund M, Schraml P, Leighton S, Torhorst J, Mihatsch MJ, Sauter G and Kallioniemi O-P (1998) Tissue microarray for high-throughput molecular profiling of tumor specimens. *Nature Med* **4**: 844–847

Kuukasjarvi T, Karhu R, Tanner M, Kahkonen M, Schaffer A, Nupponen N, Pennanen S, Kallioniemi A, Kallioniemi OP and Isola J (1997) Genetic heterogeneity and clonal evolution underlying development of asynchronous metastasis in human breast cancer. *Cancer Res* **57**: 1597–1604

Mahacek ML, Beer DG, Frank TS and Ethier SP (1993) Finite proliferative lifespan in vitro of a human breast cancer cell strain isolated from a metstatic lymph node. *Breast Cancer Res Treat* **28**: 267–276

Ram TG, Kokeny KE, Dilts CA and Ethier SP (1995) Mitogenic activity of neu differentiation factor/heregulin mimics that of epidermal growth factor and insulin-like growth factor-I in human mammary epithelial cells. *J Cell Physiol* **163**: 589–596

Ram TG, Dilts CA, Dziubinski ML, Pierce LJ and Ethier SP (1996) Insulin-like growth factor and epidermal growth factor independence in human mammary carcinoma cells with c-erbB-2 gene amplification and progressively elevated levels of tyrosine phosphorylated erbB-2. *Mol Carcinogen* **15**: 227–238

Ried T, Just KE, Holtgreve-Grez H, du Manoir S, Speicher MR, Schrock E, Latham C, Blegen H, Zetterberg A, Cremer T and Auer G (1995) Comparative genomic hybridization of formalin-fixed, paraffin-embedded breast tumors reveals different patterns of chromosomal gains and losses in fibroadenomas and diploid and aneuploid carcinomas. *Cancer Res* **55**: 5415–5423

Sartor CI, Dziubinski ML, Yu CL, Jove R and Ethier SP (1997) Role of epidermal growth factor receptor and STAT3 activation in autonomous proliferation of SUM-102PT human breast cancer cells. *Cancer Res* **57**: 978–987

Sen S, Zhou H and White RA (1997) A putative serine/threonine kinase encoding gene BTAK on chromosome 20q13 is amplified and overexpressed in human breast cancer cell lines. *Oncogene* **14**: 2195–2200

Tanner M, Tirkkonen M, Kallioniemi A, Collins C, Stokke T, Karhu R, Kowbel D, Shadrawan F, Hintz M, Kuo WL, Waldman F, Kallioniemi A, Isola J and Kallioniemi OP (1994) Increased copy number at 20q13 in breast cancer:

© 1999 Cancer Research Campaign 

defining the critical region and exclusion of candidate genes. *Cancer Res* **54**: 2457–2460

Tanner MM, Tirkkonen M, Kallioniemi A, Isola J, Kuukasjarvi T, Collins C, Kowbel D, Guan XY, Trent J, Gray JW, Meltzer P and Kallioniemi OP (1996) Independent amplification and frequent co-amplification of three nonsyntenic regions on the long arm of chromosome 20 in human breast cancer. *Cancer Res* **56**: 3441–3445

Tirkkonen M, Tanner M, Karhu R, Kallioniemi A, Isola J and Kallioniemi OP (1998) Molecular cytogenetics of primary breast cancer by CGH. *Genes Chromosomes Cancer* **21**: 177–184

Wullich B, Muller HW, Fischer U, Zang KD and Meese E (1993) Amplified met gene linked to double minutes in human glioblastomas. *Eur J Cancer* **29A**: 1991–1995

*© 1999 Cancer Research Campaign*

## References

[PDF_00611] Adnane J., Gaudray P., Dionne C. A., Crumley G., Jaye M., Schlessinger J., Jeanteur P., Birnbaum D., Theillet C. (1991). BEK and FLG, two receptors to members of the FGF family, are amplified in subsets of human breast cancers.. Oncogene.

[PDF_00615] Anzick S. L., Kononen J., Walker R. L., Azorsa D. O., Tanner M. M., Guan X. Y., Sauter G., Kallioniemi O. P., Trent J. M., Meltzer P. S. (1997). AIB1, a steroid receptor coactivator amplified in breast and ovarian cancer.. Science.

[PDF_00618] Bischoff J. R., Anderson L., Zhu Y., Mossie K., Ng L., Souza B., Schryver B., Flanagan P., Clairvoyant F., Ginther C. (1998). A homologue of Drosophila aurora kinase is oncogenic and amplified in human colorectal cancers.. EMBO J.

[PDF_00622] Collins C., Rommens J. M., Kowbel D., Godfrey T., Tanner M., Hwang S. I., Polikoff D., Nonet G., Cochran J., Myambo K. (1998). Positional cloning of ZNF217 and NABC1: genes amplified at 20q13.2 and overexpressed in breast carcinoma.. Proc Natl Acad Sci U S A.

[PDF_00629] Devilee P., Cornelisse C. J. (1994). Somatic genetic changes in human breast cancer.. Biochim Biophys Acta.

[PDF_00631] Ethier S. P. (1996). Human breast cancer cell lines as models of growth regulation and disease progression.. J Mammary Gland Biol Neoplasia.

[PDF_00637] Ethier S. P., Mahacek M. L., Gullick W. J., Frank T. S., Weber B. L. (1993). Differential isolation of normal luminal mammary epithelial cells and breast cancer cells from primary and metastatic sites using selective media.. Cancer Res.

[PDF_00633] Ethier S. P., Summerfelt R. M., Cundiff K. C., Asch B. B. (1991). The influence of growth factors on the proliferative potential of normal and primary breast cancer-derived human breast epithelial cells.. Breast Cancer Res Treat.

[PDF_00641] Fischer U., Müller H. W., Sattler H. P., Feiden K., Zang K. D., Meese E. (1995). Amplification of the MET gene in glioma.. Genes Chromosomes Cancer.

[PDF_00646] Forozan F., Karhu R., Kononen J., Kallioniemi A., Kallioniemi O. P. (1997). Genome screening by comparative genomic hybridization.. Trends Genet.

[PDF_00649] Garcia R., Yu C. L., Hudnall A., Catlett R., Nelson K. L., Smithgall T., Fujita D. J., Ethier S. P., Jove R. (1997). Constitutive activation of Stat3 in fibroblasts transformed by diverse oncoproteins and in breast carcinoma cells.. Cell Growth Differ.

[PDF_00653] Geradts J., Wilson P. A. (1996). High frequency of aberrant p16(INK4A) expression in human breast cancer.. Am J Pathol.

[PDF_00655] Guan X. Y., Meltzer P. S., Dalton W. S., Trent J. M. (1994). Identification of cryptic sites of DNA sequence amplification in human breast cancer by chromosome microdissection.. Nat Genet.

[PDF_00658] Houldsworth J., Cordon-Cardo C., Ladanyi M., Kelsen D. P., Chaganti R. S. (1990). Gene amplification in gastric and esophageal adenocarcinomas.. Cancer Res.

[PDF_00663] Kallioniemi A., Kallioniemi O. P., Piper J., Tanner M., Stokke T., Chen L., Smith H. S., Pinkel D., Gray J. W., Waldman F. M. (1994). Detection and mapping of amplified DNA sequences in breast cancer by comparative genomic hybridization.. Proc Natl Acad Sci U S A.

[PDF_00667] Karhu R., Kähkönen M., Kuukasjärvi T., Pennanen S., Tirkkonen M., Kallioniemi O. (1997). Quality control of CGH: impact of metaphase chromosomes and the dynamic range of hybridization.. Cytometry.

[PDF_00670] Kononen J., Bubendorf L., Kallioniemi A., Bärlund M., Schraml P., Leighton S., Torhorst J., Mihatsch M. J., Sauter G., Kallioniemi O. P. (1998). Tissue microarrays for high-throughput molecular profiling of tumor specimens.. Nat Med.

[PDF_00674] Kuukasjärvi T., Karhu R., Tanner M., Kähkönen M., Schäffer A., Nupponen N., Pennanen S., Kallioniemi A., Kallioniemi O. P., Isola J. (1997). Genetic heterogeneity and clonal evolution underlying development of asynchronous metastasis in human breast cancer.. Cancer Res.

[PDF_00678] Mahacek M. L., Beer D. G., Frank T. S., Ethier S. P. (1993). Finite proliferative lifespan in vitro of a human breast cancer cell strain isolated from a metastatic lymph node.. Breast Cancer Res Treat.

[PDF_00685] Ram T. G., Dilts C. A., Dziubinski M. L., Pierce L. J., Ethier S. P. (1996). Insulin-like growth factor and epidermal growth factor independence in human mammary carcinoma cells with c-erbB-2 gene amplification and progressively elevated levels of tyrosine-phosphorylated p185erbB-2.. Mol Carcinog.

[PDF_00681] Ram T. G., Kokeny K. E., Dilts C. A., Ethier S. P. (1995). Mitogenic activity of neu differentiation factor/heregulin mimics that of epidermal growth factor and insulin-like growth factor-I in human mammary epithelial cells.. J Cell Physiol.

[PDF_00689] Ried T., Just K. E., Holtgreve-Grez H., du Manoir S., Speicher M. R., Schröck E., Latham C., Blegen H., Zetterberg A., Cremer T. (1995). Comparative genomic hybridization of formalin-fixed, paraffin-embedded breast tumors reveals different patterns of chromosomal gains and losses in fibroadenomas and diploid and aneuploid carcinomas.. Cancer Res.

[PDF_00694] Sartor C. I., Dziubinski M. L., Yu C. L., Jove R., Ethier S. P. (1997). Role of epidermal growth factor receptor and STAT-3 activation in autonomous proliferation of SUM-102PT human breast cancer cells.. Cancer Res.

[PDF_00697] Sen S., Zhou H., White R. A. (1997). A putative serine/threonine kinase encoding gene BTAK on chromosome 20q13 is amplified and overexpressed in human breast cancer cell lines.. Oncogene.

[PDF_00709] Tanner M. M., Tirkkonen M., Kallioniemi A., Isola J., Kuukasjärvi T., Collins C., Kowbel D., Guan X. Y., Trent J., Gray J. W. (1996). Independent amplification and frequent co-amplification of three nonsyntenic regions on the long arm of chromosome 20 in human breast cancer.. Cancer Res.

[PDF_00714] Tirkkonen M., Tanner M., Karhu R., Kallioniemi A., Isola J., Kallioniemi O. P. (1998). Molecular cytogenetics of primary breast cancer by CGH.. Genes Chromosomes Cancer.

[PDF_00717] Wullich B., Müller H. W., Fischer U., Zang K. D., Meese E. (1993). Amplified met gene linked to double minutes in human glioblastoma.. Eur J Cancer.

